# NCES: A Cell-Specific Network-Augmented Essentiality Framework for Cancer Therapeutic Target Discovery

**DOI:** 10.34133/csbj.0160

**Published:** 2026-07-17

**Authors:** Jinmyung Jung, Sunyong Yoo

**Affiliations:** ^1^ Division of Data Science, College of Information and Communication Technology Convergence, The University of Suwon, Hwaseong 18323, Republic of Korea.; ^2^ Department of Intelligent Electronics and Computer Engineering, Chonnam National University, Gwangju 61186, Republic of Korea.; ^3^ R&D Center, MATILO AI Inc., Gwangju, Republic of Korea.

## Abstract

Identifying effective therapeutic targets remains a central challenge in cancer research. CRISPR–Cas9 knockout screens have provided valuable insights into gene essentiality; however, using essentiality at the level of individual genes often fails to reliably distinguish true therapeutic targets from nonfunctional candidates. To address this limitation, we developed the neighbor-correlation essentiality score (NCES), a network-augmented framework that leverages the essentialities of functionally active neighboring genes. NCES combines DepMap CERES scores, which estimate gene essentiality from CRISPR–Cas9 knockout screens, with protein–protein interaction networks. Interaction weights are assigned to network neighbors based on cell-line-specific expression correlations derived from CRISPR knockout or compound-perturbation profiles. The proposed NCES framework was systematically evaluated across 7 cancer cell lines against therapeutic target gold standards. NCES variants consistently outperformed approaches based solely on individual gene essentiality, with the CRISPR-weighted variant achieving the best performance, yielding AUROCs of 0.794 and 0.779 against the Therapeutic Target Database and DrugBank gold standards, respectively. Statistical testing demonstrated that weighted NCES variants significantly improved predictive accuracy over their unweighted counterpart. Finally, several high-ranking genes beyond current gold-standard datasets, including *CCNB1*, *CDC7*, and *WEE1*, were supported by the existing literature as biologically essential or therapeutically actionable. Together, these results demonstrate that NCES advances therapeutic target discovery by leveraging cell-specific, functionally relevant interactions among network neighbors within genome-scale essentiality data.

## Introduction

Identifying therapeutic molecular targets is a critical step in developing effective cancer treatments. Recently, advances in functional genomics have enabled genome-wide CRISPR–Cas9 knockout screens to systematically quantify gene essentiality. Gene essentiality is commonly measured using DepMap CERES scores, which estimate the effect of gene knockout on cell viability while correcting for copy-number-related biases [1,2]. Genes with high essentiality are often considered attractive therapeutic targets because their disruption can substantially impair cancer cell survival. Consequently, prioritizing highly essential genes has become a widely adopted strategy for therapeutic target discovery [3]. However, these essentiality-based approaches often yield limited predictive performance for therapeutic targets [4].

One way to reduce such false positives is to consider not only the essentiality of a gene itself but also that of its interacting neighbors. This concept is based on the premise that therapeutically or biologically meaningful functions rarely arise from a single gene in isolation, but rather from the coordinated activities of multiple genes within the same molecular pathway or process [5,6]. Thus, genes whose surrounding network neighbors are collectively highly essential are more likely to reflect genuinely essential biological processes, enabling more accurate predictions of therapeutic targets. However, to fully leverage this idea, it is important to account for cell-line-specific functional interactions, as the activity of molecular interactions varies substantially across cell types [7,8]. Reliance on static network structures alone may introduce nonfunctional or misleading neighbors, whereas incorporating cell-line-specific, functionally active interactions can provide a more accurate representation of underlying biological dependencies and improve prediction precision.

Previous studies have explored network-based approaches for identifying essential genes and therapeutic targets, often leveraging protein–protein interaction (PPI) networks and topological features such as degree or centrality [9,10]. More recent studies have further incorporated neighborhood-informed scoring frameworks that integrate gene essentiality with the network context to improve prediction performance [11]. In addition, large-scale CRISPR–Cas9 screening data have been used to characterize gene essentiality and to infer functional gene modules based on coessentiality patterns [12,13]. However, these methods typically rely on static network structures or treat gene essentiality as an individual property, without explicitly modeling the functional influence of neighbor genes in a cell-line-specific manner.

To address these limitations, the present study offers several key contributions. First, the proposed neighbor-correlation essentiality score (NCES) framework models the influence of interacting genes by aggregating neighbor essentiality, moving beyond individual gene-level analysis. Second, NCES incorporates cell-line-specific functional interactions by weighting network neighbors using perturbation-derived expression correlations, enabling cell-line-specific modeling of gene dependencies. Third, the framework is designed and systematically evaluated for therapeutic target prediction, a challenging and translationally relevant task, using independently constructed gold-standard datasets across multiple cancer cell lines.

**Fig. 1.**
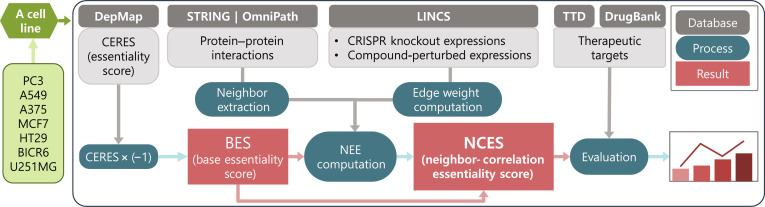
Strategy overview. For each of the 7 cancer cell lines, CERES scores from DepMap were inverted to obtain the base essentiality score (BES). The neighbors of each protein were identified using protein–protein interaction networks from the Search Tool for the Retrieval of Interacting Genes/Proteins (STRING) or the OmniPath database. Edge weights to neighbors were estimated by incorporating CRISPR knockout or compound-perturbed expression profiles from the Library of Integrated Network-based Cellular Signatures (LINCS) database. These weighted neighbor effects were integrated with the associated BES to compute the neighbor essentiality effects (NEEs), which were subsequently used to derive the neighbor-correlation essentiality score (NCES). The predictive performance of NCES was evaluated against known therapeutic targets obtained from the Therapeutic Target Database (TTD) and DrugBank. All analyses were conducted using cell-line-specific DepMap and LINCS data together with cancer-subtype-specific therapeutic target annotations derived from TTD and DrugBank.

In this study, we developed NCES, a network-augmented framework that integrates intrinsic essentiality with correlation-weighted neighbor effects (Fig. 1). NCES combines DepMap CERES scores with PPI networks and weights neighbor contributions using cell-line-specific, perturbation-derived correlations. The framework was systematically evaluated across 7 cancer cell lines using 2 therapeutic target gold standards. This approach enables more accurate prioritization of therapeutically relevant genes by capturing cell-line-specific functional dependencies among interacting genes.

## Materials and Methods

### Baseline essentiality score

CERES scores were retrieved from the CRISPR_Gene_Effect file in the DepMap database (release 24Q4) for 7 cell lines with available data: PC3 (prostate cancer), A549 (lung cancer), A375 (melanoma), MCF7 (breast cancer), HT29 (colorectal cancer), BICR6 (head and neck cancer), and U251MG (glioblastoma). The CERES score is derived from genome-wide CRISPR–Cas9 knockout screens that quantify the effects of gene perturbation on cell growth and further incorporates a computational correction for copy-number-related biases to more accurately estimate gene essentiality [2]. The CERES score is defined such that more negative values indicate higher essentiality. Accordingly, the base essentiality score (BES) was obtained by multiplying the CERES score by −1, thereby converting the score into a positively scaled metric where higher values reflect stronger essentiality. Consequently, the A549, A375, MCF7, HT29, and U251MG cell lines contained BES values for 17,916 genes, whereas the PC3 and BICR6 cell lines contained BES values for 17,787 genes.

### Neighbor-correlation essentiality score

#### Construction of the PPI networks

To construct the PPI networks, 2 distinct databases were independently utilized, which exhibit different characteristics: Search Tool for the Retrieval of Interacting Genes/Proteins (STRING; v12.0) and OmniPath (accessed in March 2025). The STRING database provides a comprehensive collection of protein associations, offering extensive coverage across the human proteome [14]; however, this database includes a subset of low-confidence or indirect associations inferred from text mining and coexpression data. Thus, we extracted 235,130 PPIs involving 15,970 proteins from the STRING database, using a combined score threshold of 700.

The OmniPath database primarily contains high-confidence, manually curated interactions compiled from multiple signaling resources [15]. From the OmniPath database, 183,644 interactions among 16,394 proteins were retrieved. Both datasets were represented as undirected graphs, which were then used in parallel analyses to evaluate their predictive performance for therapeutic targets.

#### Expression profiles used for edge weight computation

We used 2 distinct expression profile types from the Library of Integrated Network-based Cellular Signatures (LINCS) database (dataset LDS-1613; CMap L1000 Phase 3, Level 5, accessed in March 2025) to compute edge weights for PPIs. Firstly, genome-wide expression profiles induced by CRISPR–Cas9 knockout screening were obtained from the LINCS database for each selected cell line [16]. Each gene was individually knocked out using CRISPR–Cas9, and the expression levels of 12,328 genes were measured 72 h after knockout. If multiple knockout experiments were available for the same gene, their averages were used. The number of genes knocked out in each cell line was as follows: PC3 (5,009), A549 (5,020), A375 (5,014), MCF7 (4,183), HT29 (5,009), BICR6 (5,009), and U251MG (5,009).

Secondly, we obtained genome-wide expression profiles perturbed by an extensive set of compound treatments from the LINCS database [16]. Each profile comprised the expression levels of 12,328 genes measured after compound exposure. Among the various experimental settings available in LINCS, we selected the most abundant condition—treatment with a 10 μM dose for 24 h—to ensure data consistency and comparability across cell lines. When replicate profiles were available for the same compound, expression values were averaged. The number of compounds treated per cell line was as follows: PC3 (8,703), A549 (6,742), A375 (4,740), MCF7 (7,975), HT29 (4,109), BICR6 (108), and U251MG (112).

#### Formulation of NCES

The NCES of a gene *G*, i.e., *NCES*(*G*), is defined as follows:

Take

G:gene of interest

$$N_{i}:i\text{th}\;\text{neighbor gene of}\;G\;\left(i=1,\ldots ,m\right)$$

$$\begin{array}{c} \begin{array}{c} \begin{array}{c} \mathrm{cor}(G,\;N_{i}):\text{Pearson correlation between the expression profiles of}\;G\;\text{and}\;N_{i} \end{array} \end{array} \end{array}$$

Define

$$w(G,N_{i})={\text{exp}}^{\left|\text{cor}(G,\;N_{i})\right|}$$ (1)

$$\text{NWE}\left(G\right)={\left(\text{BES}\left(N_{i}\right)\cdot w(G,\;N_{i})\right)}_{i=1}^{m}$$ (2)

$$\text{NEE}\left(G\right)=\text{max}\;\left(\mathrm{NWE}\left(G\right)\right)$$ (3)

$$\begin{array}{c} \text{NCES}\left(G\right)=\left(1-p\right)\cdot \text{BES}\left(G\right)+p\cdot \text{NEE}\left(G\right) \end{array}$$ (4)

where *w* is the edge weight, *NWE*(*G*) denotes the neighbor-weighted essentiality score of gene *G*, *NEE*(*G*) is the neighbor essentiality effect of gene *G*, and *p* is the neighbor effect scaling factor.

In this study, 2 distinct types of correlations were considered. First, protein–protein correlations were quantified using the Pearson correlations derived from CRISPR-knockout-induced expression profiles. Each profile consisted of genome-wide expression measurements across 12,328 genes, obtained after individually knocking out the corresponding gene. This genome-wide perturbational signature captures the transcriptional consequences of gene loss. Based on these correlations, we derived an NCES variant, hereafter referred to as the CRISPR-weighted NCES (NCES CRISPR).

Second, protein–protein correlations were quantified using Pearson correlations derived from compound-perturbed expression profiles. For each gene, the profile consisted of its expression levels measured across multiple compound-perturbation conditions. In contrast, CRISPR-derived profiles captured genome-wide transcriptional responses induced by the knockout of each gene. The number of treated compounds varied across cell lines (e.g., 8,703 compounds for PC3 versus 112 for U251MG). Based on these correlations, we derived an NCES variant, hereafter referred to as the compound-weighted NCES (NCES Comp). In both cases, correlations were calculated using cell-specific expression profiles.

The edge weight (*w*) was finally defined as the exponential transformation of the absolute-correlation value, yielding a weight range of 1 ≤ *w* ≤ *e* (≒2.718). When the expression profile for either of the 2 proteins was unavailable, a default correlation value of 0 was assigned, yielding an edge weight of 1. The aggregation function was set to the maximum, as alternatives such as average or median yielded inferior performance (see the ablation study). This choice reflects the assumption that a gene’s functional importance may be dominated by its most critical interaction partner. The NCES was computed using 4 values of the neighbor effect scaling factor *p*: 0.2, 0.4, 0.6, and 0.8.

To evaluate the effect of cell-specific correlations on predictive performance, we additionally constructed an unweighted NCES scoring scheme, referred to as the base NCES (NCES Base). In this scheme, all correlation terms in the above formulation were set to 0, such that all edge weights were fixed to 1. NCES Base, which excludes all cell-specific correlation information, serves as a baseline for assessing the impact of correlation-informed scoring.

### Evaluation strategy

#### Construction of cancer-subtype-specific gold-standard sets

To evaluate the proposed framework, 2 types of therapeutic target sets were independently constructed as gold standards for each of the 7 analyzed cell lines.

The first therapeutic target sets were obtained from the Therapeutic Target Database (TTD; accessed in March 2025), which provides comprehensive information on known/potential therapeutic targets and associated diseases [17]. In the TTD, the disease-related field for each therapeutic target specifies cancer subtype names rather than cell line names. Accordingly, a cell-line-to-cancer subtype mapping table was used to extract cancer-subtype-specific therapeutic target lists (Table S1). When the disease field in the TTD was annotated as “solid tumor/cancer”, the corresponding therapeutic targets were included for all 7 analyzed cell lines, as these targets are all derived from solid tumors. To ensure a more reliable construction of the gold-standard set, only therapeutic targets that had reached phase II clinical development or a more advanced regulatory status were included (i.e., phase 2, phase 2a, phase 2b, phase 2/3, phase 3, phase 4, New Drug Application filed, Biologics License Application submitted, application submitted, approval submitted, preregistration, registered, and approved). The extracted therapeutic target lists are provided in Table S2.

The second therapeutic target set was obtained from DrugBank (accessed in March 2025), a comprehensive database that contains detailed information on drugs, including associated indications and corresponding molecular targets [18]. Similar to the TTD, the “indications” field provides cancer subtype information rather than cell line annotations. Therefore, a cell-line-to-cancer subtype mapping table (Table S1) was used to extract cancer-subtype-specific therapeutic target lists (Table S2). To further assess the overlap among the resulting gold-standard sets, pairwise Jaccard indices were calculated across the 7 analyzed cancer subtypes, and the results are summarized in Table S3.

#### AUROC calculation

The performance of NCES was quantitatively evaluated using the area under the receiver operating characteristic curve (AUROC) curve [19]. The AUROC curve measures the discriminative ability of a scoring system to distinguish between positive and negative instances, providing a threshold-independent indicator of classification performance. For each analyzed cell line, the therapeutic targets curated from the TTD and DrugBank were used as positive instances, whereas all remaining genes were treated as negative instances. False-positive and true-positive rates were computed to construct the receiver operating characteristic (ROC) curves using the roc_curve and auc functions in the scikit-learn library.

## Results

### Gene essentiality scoring using 3 NCES variants

**Fig. 2.**
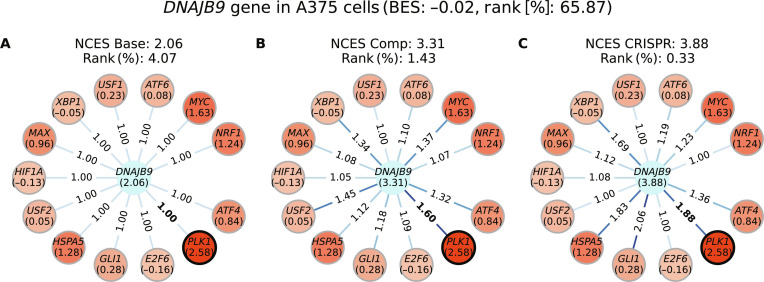
Network-based illustration of the 3 neighbor-correlation essentiality score (NCES) variants for the *DNAJB9* gene in A375. Panels show the essentiality score (cyan central node) of *DNAJB9* based on (A) NCES Base, (B) NCES Comp, and (C) NCES CRISPR. Neighboring genes are shown in orange, with their base essentiality score (BES) values indicated in the nodes, while edges are represented by blue lines, with numbers indicating the edge weights with the *DNAJB9* gene. Across all 3 NCES variants, *PLK1* contributes the maximum neighbor essentiality effect (NEE) among neighboring genes, with NEE values of (A) 2.58, (B) 4.14, and (C) 4.85. Consequently, the NCES of *DNAJB9* is (A) 2.06 (top 4.07%), (B) 3.31 (top 1.43%), and (C) 3.88 (top 0.33%). All NCES values shown were computed using the OmniPath-based protein–protein interaction (PPI) network and *p* = 0.8.

For each of the 7 analyzed cell lines (PC3, A549, A375, MCF7, HT29, BICR6, and U251MG), the 3 NCES variants (NCES Base, NCES Comp, and NCES CRISPR) were computed across all combinations of 2 PPI networks (STRING and OmniPath) and 4 values of the scaling factor *p* (0.2, 0.4, 0.6, and 0.8). Figure 2 shows how the 3 NCES variants were computed for the *DNAJB9* gene in A375 cells. In this example, NCES CRISPR markedly elevated the essentiality rank of *DNAJB9* compared to the BES, illustrating how functional neighbor information can alter gene prioritization. Conversely, Fig. S1 shows an example of the *NSF* gene in MCF7 cells, whose rank decreased from 0.68% based on BES alone to approximately 51% under all NCES variants. Because its neighboring genes exhibited uniformly low essentiality scores, *NSF* received little support from the network. This example highlights that NCES can both prioritize and deprioritize genes depending on the essentiality patterns of their functional neighbors.

**Fig. 3.**
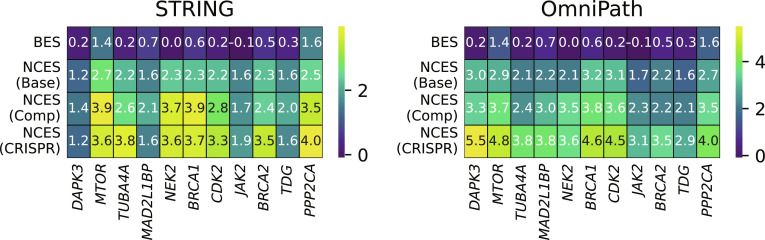
Heatmaps of base essentiality score (BES) and the 3 neighbor-correlation essentiality score (NCES) variants for selected genes in the A375 cell line across the Search Tool for the Retrieval of Interacting Genes/Proteins (STRING) and OmniPath protein–protein interaction (PPI) networks. For each selected gene, essentiality scores of BES, NCES Base, NCES Comp, and NCES CRISPR are depicted. The heatmaps illustrate how gene essentiality scores and rankings vary across different network choices and NCES variants. All NCES values shown were computed using a neighbor-effect scaling factor of *p* = 0.8.

Additionally, Fig. 3 illustrates heatmaps of BES and the 3 NCES variants for selected genes, enabling comparisons of essentiality scores across genes under different NCES variants and network choices. For instance, within the OmniPath-based network, *DAPK3* exhibits a substantially lower BES (0.2) than *PPP2CA* (1.6); however, under NCES CRISPR, *DAPK3* attains a markedly higher score (5.5) than *PPP2CA* (4.0). This example highlights that the proposed NCES framework can substantially reshape relative gene essentiality rankings by incorporating network-derived, correlation-weighted neighbor effects.

### Therapeutic target predictive performance

**Fig. 4.**
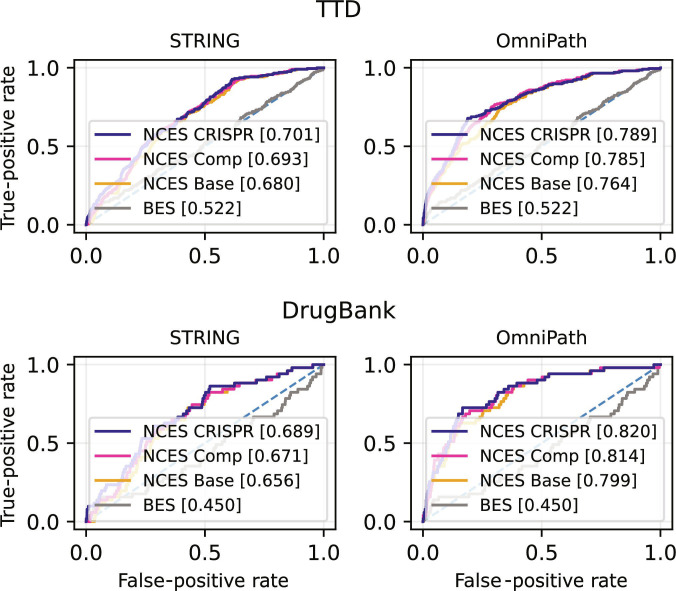
Receiver operating characteristic (ROC) curve of 4 gene essentiality scoring schemes in the A375 cell line. Curves are shown for the 4 scoring schemes—base essentiality score (BES), neighbor-correlation essentiality score (NCES) Base, NCES Comp, and NCES CRISPR—evaluated against 2 therapeutic target gold standards (Therapeutic Target Database [TTD] and DrugBank). For each panel, the corresponding area under the ROC curve (AUROC) value is indicated in brackets. All NCES scores were computed using a neighbor-effect scaling factor of *p* = 0.8.

To evaluate the predictive performance in therapeutic target identification, 4 scoring systems (BES, NCES Base, NCES Comp, and NCES CRISPR) were evaluated by computing AUROC curves against 2 therapeutic target gold standards (TTD and DrugBank) for each cell line. For example, the ROC curves for the A375 cell line across the 2 PPI networks are illustrated in Fig. 4, where NCES was computed using a scaling factor of *p* = 0.8. Across all comparisons, the 3 NCES variants outperform BES, with NCES CRISPR achieving the highest AUROC values. Moreover, NCES based on the OmniPath PPI network yields higher AUROC values than its STRING-based counterpart.

**Fig. 5.**
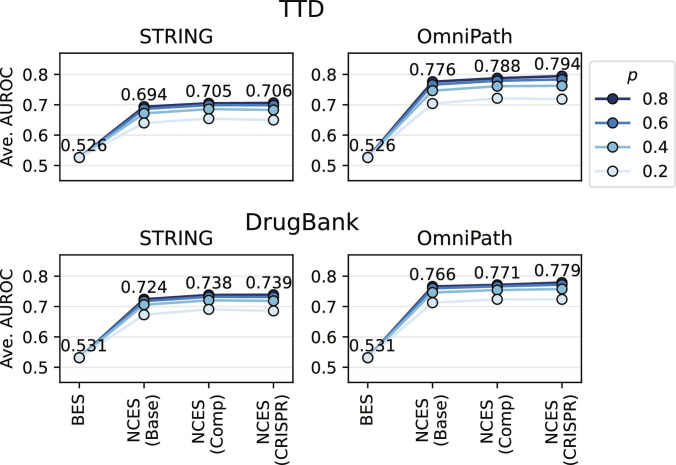
Performance comparison of 4 gene essentiality scoring schemes. Four gene essentiality scoring schemes were evaluated using 2 therapeutic target gold standards (Therapeutic Target Database [TTD] and DrugBank). For each scoring scheme, the plotted points represent the mean area under the receiver operating characteristic curve (AUROC) computed across 7 cancer cell lines. The evaluations were performed across 2 protein–protein interaction (PPI) networks (Search Tool for the Retrieval of Interacting Genes/Proteins [STRING] and OmniPath) and 4 neighbor-effect scaling parameters *p* (0.2, 0.4, 0.6, and 0.8). AUROC values shown as text in each panel correspond to the mean AUROC calculated at *p* = 0.8. BES, base essentiality score; NCES, neighbor-correlation essentiality score.

The AUROC results for NCES across the 7 cell lines, computed using 4 neighbor-effect scaling factors (*p* = 0.2, 0.4, 0.6, and 0.8), are provided in Table S4. These results were summarized by computing the mean AUROC curves across the 7 cell lines for each scoring scheme, PPI network, and neighbor-effect scaling parameter *p* in Fig. 5. Consistent with the trends observed in Fig. 4, the NCES variants consistently and substantially outperformed BES across both gold standards. These findings indicate that incorporating neighbor essentiality yields markedly higher predictive accuracy than relying solely on gene intrinsic essentiality. Among the NCES variants, NCES CRISPR and NCES Comp achieved higher AUROC values than NCES Base across most settings, indicating that the incorporation of cell-line-specific edge weights improves predictive performance.

In addition, NCES CRISPR consistently shows slightly better performance than NCES Comp. This improvement may well reflect that genome-wide transcriptional responses induced by gene knockout more directly capture the functional impact of gene perturbation. Moreover, models based on the OmniPath network achieved higher AUROCs than those based on STRING, highlighting the advantage of higher-confidence, manually curated interactions. Finally, performance showed a slight but consistent increase with larger *p* values, suggesting that assigning greater influence to neighbor essentiality enhances predictive accuracy.

**Fig. 6.**
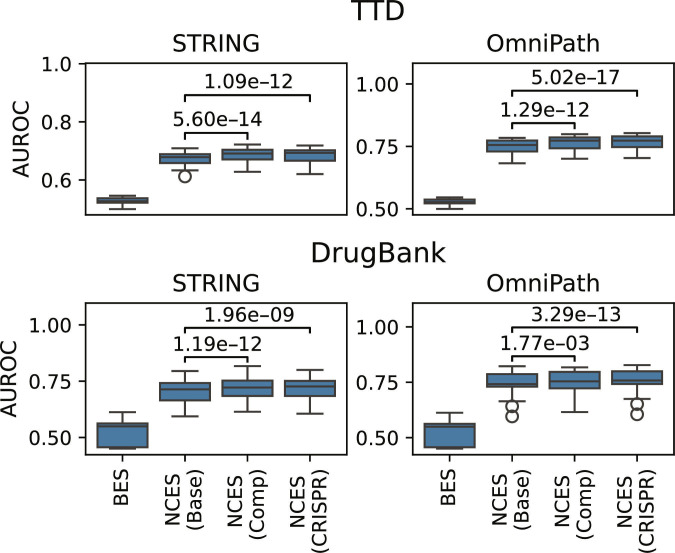
Statistical comparison of area under the receiver operating characteristic curve (AUROC) values across neighbor-correlation essentiality score (NCES) scoring schemes. Boxplots illustrate the AUROC distributions for the 4 essentiality score schemes across 7 cell lines and 4 *p* values (28 instances per box). One-sided paired *t* tests were used to compare NCES Base with the 2 cell-specific-weighted variants (NCES Comp and NCES CRISPR). TTD, Therapeutic Target Database; BES, base essentiality score.

To assess whether the differences in predictive performance among the NCES variants were statistically significant, we conducted pairwise comparisons of the associated AUROC values. We compared NCES Base with the 2 cell-specific weighted variants (NCES Comp and NCES CRISPR) using one-sided paired *t* tests. A total of 28 paired instances were used in each comparison, corresponding to 7 cell lines evaluated across 4 values of the neighbor-effect scaling parameter *p*. Across all comparisons, the 2 cell-specific-weighted NCES variants consistently achieved significantly higher AUROCs than NCES Base (*P* < 0.005) (Fig. 6).

When integrating all evaluation results, the NCES CRISPR model under the OmniPath-based network with a neighbor-effect scaling factor of *p* = 0.8 achieved the strongest overall performance, yielding average AUROCs of 0.794 and 0.779 for TTD and DrugBank, respectively. The final essentiality scores derived under this optimal configuration are summarized in Table S5.

### Robustness and additional validation analyses

To further assess the robustness of the selected neighbor-effect scaling factor, we performed leave-one-cell-line-out cross-validation (LOOCV) across the 7 cell lines. In each fold, the optimal value of *p* (0.2, 0.4, 0.6, or 0.8) was selected using the remaining 6 cell lines and evaluated on the held-out cell line. The LOOCV results were highly consistent with those obtained in the primary analysis. Notably, *p* = 0.8 was selected as the optimal scaling factor in all validation folds, indicating that the parameter selection was highly stable across different training sets. Furthermore, the cross-validation performance closely matched that of the primary analysis, suggesting that the selection of *p* = 0.8 was not driven by overfitting. Detailed LOOCV results are provided in Table S6.

Furthermore, we augmented Table S6 with druggability annotations obtained from the Pharos database [20]. Pharos classifies targets according to their Target Development Level (TDL), reflecting the extent of pharmacological and clinical evidence available for each gene. We collected all genes annotated as Tclin (targets of approved drugs) or Tchem (targets with known bioactive small molecules), resulting in 2,608 druggable genes. For each gene, we additionally provide its Illuminating the Druggable Genome (IDG) family classification, including enzymes (*n* = 886), kinases (*n* = 442), G protein-coupled receptors (*n* = 261), ion channels (*n* = 211), and other target classes. When AUROC analyses were repeated using only the druggable genome, the overall performance trends remained highly consistent with the primary results, with NCES CRISPR continuing to achieve the strongest predictive performance (Fig. S2).

To further evaluate the biological relevance of the cell-specific weighting scheme, we examined gold-standard genes (GS genes) whose rankings were substantially improved by NCES CRISPR compared with NCES Base (rank improvement >10% and final NCES CRISPR rank within the top 5%). The complete list of genes meeting these criteria is provided in Table S7. Representative examples with established therapeutic relevance in the corresponding cancer types, including *ALK* in A375 cells, *CXCR4* and *JAK2* in A549 cells, *FGFR3* in HT29 cells, and *PARP1* in MCF7 cells, are highlighted in Table 1. These findings suggest that incorporating cell-specific perturbation correlation information can prioritize cancer-context-specific therapeutic targets that are less prominent in the unweighted NCES Base model.

**Table 1.** Representative gold-standard genes substantially prioritized by NCES CRISPR relative to NCES Base under the optimal configuration (OmniPath network, *p* = 0.8). Genes were selected based on a rank improvement >10% and a final NCES CRISPR rank within the top 5%.

GS	Cell	Gene	Base rank (%)	CRISPR rank (%)	ΔRank (%)	Cancer relevance
TTD	A375	*ALK*	24.93	4.24	20.69	Melanoma-associated receptor tyrosine kinase signaling [36]
TTD	HT29	*FGFR3*	16.2	3.19	13.01	FGFR signaling implicated in colorectal cancer progression [37]
TTD	A549	*JAK2*	17.03	4.51	12.52	JAK/STAT signaling associated with NSCLC growth and survival [38]
DrugBank	A549	*CXCR4*	17.26	3.4	13.85	Promotes lung cancer invasion and metastasis through CXCL12–CXCR4 signaling [39]
DrugBank	MCF7	*PARP1*	12.1	1.99	10.11	Clinically relevant DNA repair target in breast cancer [40]

NCES, neighbor-correlation essentiality score; GS, gold standard; TTD, Therapeutic Target Database; FGFR, fibroblast growth factor receptor; JAK, Janus kinase; STAT, signal transducer and activator of transcription; NSCLC, non-small cell lung cancer

To evaluate whether the predictive performance of NCES was driven by broadly essential genes, we repeated the AUROC analysis after removing 1,827 common essential genes from the evaluation set, based on the DepMap common essential genes. Despite the removal of these genes, the overall performance trends remained highly consistent with the primary analysis (Fig. S3). In the results, NCES CRISPR continued to achieve the highest predictive performance, followed by NCES Comp and NCES Base. The overall performance trends remained unchanged after excluding common essential genes, indicating that the predictive performance of NCES is not primarily driven by these genes.

Because NCES CRISPR incorporates edge weights derived from LINCS CRISPR perturbation data, we further investigated whether the observed performance improvement could be influenced by differential LINCS coverage between GS genes and non-gold-standard (non-GS) genes. We first quantified the proportion of genes with available LINCS CRISPR knockout experiments in each cell line (Table S8). GS genes showed substantially higher coverage than non-GS genes across all cell lines, with approximately 64% to 87% of GS genes having corresponding LINCS perturbation experiments compared with only 22% to 27% of non-GS genes. To assess the impact of this potential bias, we repeated the evaluation under 2 additional settings: (a) restricting the evaluation to genes with available LINCS CRISPR knockout experiments and (b) restricting the evaluation to genes without available LINCS CRISPR knockout experiments. As shown in Fig. S4, NCES consistently outperformed BES in both settings. These results demonstrate that the predictive signal captured by NCES is not solely driven by the availability of direct LINCS perturbation experiments and can be propagated through neighboring genes in the interaction network.

To assess the potential influence of discovery bias in contemporary PPI databases, we repeated the analysis using historical STRING networks, including v10 (April 2016), v10.5 (May 2017), and v11.0 (January 2019). As shown in Fig. S5, all NCES variants consistently outperformed BES across all historical STRING versions, while the correlation-weighted variants (NCES CRISPR and NCES Comp) maintained superior performance over the unweighted NCES Base model. These results suggest that the predictive utility of NCES is not solely driven by contemporary knowledge embedded in current PPI databases.

### Gene set enrichment analysis

Gene set enrichment analysis (GSEA) is a computational method that assesses whether predefined gene sets exhibit statistically significant enrichment within a ranked list of genes [21]. To perform GSEA, a representative NCES profile was constructed by aggregating NCES values across the 7 analyzed cell lines under the optimal configuration. The robust rank aggregation method [22] was used for this aggregation. The representative NCES profiles are provided in Table S9, together with their TDL and IDG family annotations from the Pharos database. The profile was used as input for GSEA, which was performed using the “GO_Biological_Process_2023” gene set with the GSEApy package in Python. A total of 527 significantly enriched biological processes (normalized enrichment score > 0, false discovery rate *q* value < 0.05) are listed in Table S10. The top 10 enriched processes predominantly represent essential biological functions, including DNA damage response, cell cycle regulation, and DNA replication (Table 2).

**Table 2.** Top 10 significantly enriched biological processes from GSEA

Rank	Biological process
1	Regulation of the meiotic cell cycle (GO:0051445)
2	Anaphase-promoting complex-dependent catabolic process (GO:0031145)
3	Regulation of ubiquitin protein ligase activity (GO:1904666)
4	Nuclear transport (GO:0051169)
5	Mitotic G1 DNA damage checkpoint signaling (GO:0031571)
6	Positive regulation of miRNA transcription (GO:1902895)
7	Protein K11-linked ubiquitination (GO:0070979)
8	DNA strand elongation involved in DNA replication (GO:0006271)
9	Positive regulation of mitotic sister chromatid separation (GO:1901970)
10	Positive regulation of chromosome segregation (GO:0051984)

GSEA, gene set enrichment analysis; miRNA, microRNA

### Ablation study

**Fig. 7.**
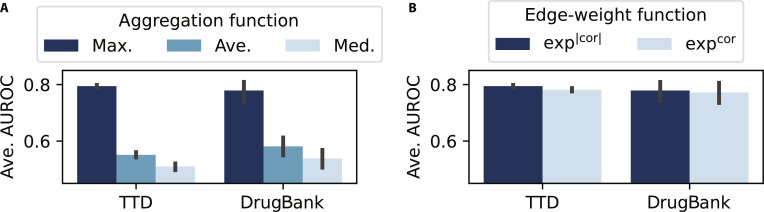
Ablation analysis of aggregation and edge weight functions in the neighbor-correlation essentiality score (NCES) framework. (A) A comparison of 3 aggregation functions, i.e., maximum, average, and median, within the final NCES model (NCES CRISPR on the OmniPath network with *p* = 0.8). Predictive performance was evaluated against 2 therapeutic target gold standards (Therapeutic Target Database [TTD] and DrugBank). (B) A comparison of 2 edge-weight functions, i.e., exp(|cor|) and exp(cor), within the final NCES model.

To evaluate the contribution of individual design choices, we performed an ablation study using the best-performing NCES variant (i.e., the NCES CRISPR constructed on the OmniPath network with *p* = 0.8) as the reference model. First, we replaced the aggregation function, max (max.), with either average (ave.) or median (med.). The subsequent predictive performance was then assessed against the 2 therapeutic target gold standards (Fig. 7A). The results show that in the TTD benchmark, the mean AUROCs for the max, average, and median aggregation methods were 0.794, 0.551, and 0.510, respectively, whereas the corresponding values in the DrugBank benchmark were 0.779, 0.581, and 0.538. In both cases, the max aggregation substantially outperformed the alternatives.

To better understand the behavior of the max aggregation function, we further examined the distribution of argmax neighbors, defined as the neighboring genes contributing the maximum NEE for each target gene. Although several regulators, including *MYC*, *CTCF*, and *CDK1*, appeared more frequently than expected, the distribution was not dominated by a small number of genes (Table S11). Across all 7 cell lines, the top 10, 20, 50, and 100 argmax neighbors accounted for 40.4%, 50.6%, 64.9%, and 75.5% of all argmax-neighbor occurrences, respectively. These findings suggest that the superior performance of max aggregation is not simply driven by proximity to a few hub genes. Rather, max aggregation preserves the strongest local dependency signal within a gene’s neighborhood, whereas average and median aggregation tend to dilute such signals by incorporating numerous weakly associated neighbors.

Second, we examined the effect of the edge-weight function by replacing the exponential absolute-correlation weighting, exp(|cor|), with a signed exponential weighting, exp(cor) (Fig. 7B). In the TTD benchmark, the mean AUROCs for exp(|cor|) and exp(cor) were 0.794 and 0.782, respectively, whereas in the DrugBank benchmark, the corresponding values were 0.779 and 0.772. In both cases, exp(|cor|) achieved slightly higher predictive performance.

## Discussion

### Impact of neighbor essentiality on NCES performance

Incorporating neighbor-essentiality information substantially enhanced the predictive power of the scoring scheme, as reflected by the higher AUROC values for the therapeutic target predictions. This enhancement arises because essential genes seldom function in isolation; rather, these genes operate within tightly connected molecular modules that coordinate key cellular processes such as DNA replication and cell cycle regulation. Indeed, NCES captures the contextual gene essentiality by incorporating the essentiality scores of neighboring genes within the interaction network. This finding underscores the importance of the network-level context in refining essentiality-based prioritization [23].

The neighbor essentiality information is aggregated in our formulation using a max operator, and the ablation study confirmed that this max-based approach achieves substantially better performance than aggregation by averaging or taking the median. This result likely arises because essential neighbor effects are typically driven by a small number of highly influential neighbors [6]. Consequently, a max-based operator is better suited to capturing these dominant dependencies, whereas averaging or taking the median tends to dilute the contribution of these high-impact signals.

### Benefits of incorporating perturbation-derived correlation weights into NCES

NCES variants incorporating correlation-based edge weights, such as NCES CRISPR and NCES Comp, yielded small but consistent improvements in therapeutic target prediction compared with NCES Base. Even though the performance gain was modest, the result was biologically meaningful. These edge weights, derived from cell-specific expression data, help propagate essentiality signals through pathways that are active in each cell line. This makes the model more sensitive to cell-specific interactions and better reflects how functional relationships can vary across cell lines [24]. The improvement in the AUROC values suggests that adding cell-specific molecular information increases the biological realism of the scoring framework.

Among the 2 types of evaluated correlation-based weights, the CRISPR-based correlations consistently outperformed the compound-based correlations. A likely explanation is that CRISPR perturbations cause more direct, gene-specific changes in expression. More specifically, knocking out a gene induces genome-wide transcriptional responses that reveal the causal functional consequences of losing the given gene, thereby clarifying how strongly the gene is functionally linked to any associated partners [25]. In contrast, the expression profiles generated under compound treatments were influenced by the broad effects of small molecules. Since these compounds can act on multiple pathways simultaneously and do not necessarily perturb the activity of the corresponding gene, the resulting correlations are less reliable indicators of functional coupling between interacting proteins.

Moreover, the exponential absolute-correlation weights, exp(|cor|), used in our ablation study yielded slightly better results than the signed exponential weights, exp(cor). This is likely because the strength of an interaction is determined by how strongly 2 genes respond together, regardless of whether the gene expressions are up- or down-regulated. Indeed, negative correlations can still represent meaningful functional relationships [26], and taking the absolute value preserves these informative signals, whereas the signed version would diminish or ignore these signals.

### Therapeutically relevant high-ranking genes beyond current gold standards

NCES exhibited high predictive performance, with an AUROC of approximately 0.78, thereby demonstrating the ability of NCES to distinguish known therapeutic targets from nontarget genes. Therefore, genes that ranked highly in NCES but were not included in the curated gold-standard datasets may represent biologically and therapeutically relevant candidates deserving further investigation. Thus, we identified 15 genes that ranked within the top 30 of the representative NCES profile but were not included in either of the 2 therapeutic gold-standard datasets: *CCNB1*, *SRSF2*, *TPR*, *WEE1*, *BRCA1*, *CDC7*, *PSMA7*, *U2AF1*, *PDPK1*, *CCNA2*, *UBE2I*, *CDC27*, *KMT2D*, *MCM2*, and *BUB1* (Table S9).

Among these 15 genes, several genes were selected for more in-depth literature validation. *CCNB1* (ranked second in our analysis) encodes a key regulator of the G2/M cell cycle transition. *CCNB1* overexpression has been consistently associated with aggressive tumor biology and poor patient survival across multiple solid cancers, including lung, breast, and esophageal malignancies [27]. Recent pan-cancer and disease-focused studies have further shown that *CCNB1* up-regulation correlates with adverse prognosis, lymphovascular invasion, and metastatic potential and that modulating *CCNB1* expression can affect tumor growth, invasion, and therapy sensitivity, collectively supporting *CCNB1* as a candidate prognostic biomarker and therapeutic target in several malignancies [28]. The high NCES ranking of *CCNB1* demonstrates that the framework can successfully prioritize genes with well-established biological and therapeutic relevance, even when they are not represented in the curated gold-standard datasets.

*WEE1* (ranked 12th in our analysis) encodes a serine/threonine kinase that governs the G2/M checkpoint by inhibiting CDK1/2 and thereby delaying mitotic progression. *WEE1* inhibition promotes the propagation of tumor cells with *TP53* mutations into mitosis before DNA repair is complete, making these cells highly vulnerable to mitotic failure [29]. Preclinical studies have shown that inhibiting *WEE1* makes cancer cells more sensitive to DNA-damaging chemotherapies, while clinical trials of *WEE1* inhibitors in solid tumors further support its potential as a therapeutic target [30]. These findings collectively support *WEE1* as a promising therapeutic target for cancers that rely on the G2/M checkpoint.

Furthermore, *BRCA1* (ranked 15th) is a key DNA repair gene implicated in hereditary breast and ovarian cancers, providing a strong benchmark for known oncogenic and therapeutic relevance. Loss of *BRCA1* function leads to defects in DNA repair, making tumor cells particularly sensitive to *PARP* inhibitors, which account for the clinical effectiveness of these inhibitors in *BRCA* mutant cancers. [31,32]. Additionally, *CDC7* (ranked 18th) has been reported to be overexpressed in multiple cancers, suggesting that it may be a promising target for small-molecule inhibition. Blocking *CDC7* interferes with DNA replication and leads to the death of fast-growing tumor cells, making *CDC7* an attractive target for cancer therapy [33–35].

Notably, some identified genes such as *WEE1* and *BRCA1* are well-known cancer-related genes with therapeutic relevance; however, they are not included in the gold-standard datasets. This discrepancy may reflect the conservative curation criteria of existing databases, as well as the distinction between direct drug targets and indirect therapeutic vulnerabilities. These observations suggest that NCES can capture therapeutically meaningful genes beyond the scope of existing curated resources.

### Limitations of current therapeutic target benchmarks

One limitation of this study is that the gold-standard sets were derived from DrugBank and TTD, which primarily contain therapeutic targets that have already been investigated pharmacologically. Consequently, our evaluation framework assesses the ability of NCES to prioritize currently recognized therapeutic targets rather than entirely novel disease dependencies. In addition, emerging therapeutic modalities, such as proteolysis-targeting chimeras and molecular glues, continue to expand the range of therapeutically actionable proteins beyond those represented in current databases. Therefore, while DrugBank- and TTD-based evaluations provide a clinically relevant benchmark, they may not fully capture the broader landscape of future therapeutic targets.

## Conclusion

This study introduced NCES, a cell-line-specific, network-augmented essentiality scoring framework designed to improve therapeutic target prediction. By incorporating the essentiality of functionally active neighbors, rather than relying solely on intrinsic essentiality, NCES captures therapeutically meaningful signals that traditional essentiality metrics fail to detect. A cancer-subtype-specific evaluation using independently constructed gold-standard datasets for each of the 7 cancer cell lines showed that all NCES variants markedly outperformed the BES scheme, with the CRISPR-weighted NCES achieving the highest performance. These findings highlight the importance of considering network structure as well as cell-specific functional activity when prioritizing therapeutic targets. Many top-ranked genes not represented in current gold-standard datasets were supported by independent literature as promising therapeutic candidates. Overall, by integrating multilayer functional data with the network context, the NCES provides a powerful framework for identifying therapeutic targets. Future work may extend NCES to more cancer types, incorporate multi-omics and clinical data, and adapt the framework for use in precision oncology.

## Data Availability

The data supporting the conclusions of this study are available in the paper and/or the Supplementary Materials. The datasets analyzed in this study were obtained from publicly available resources, including DepMap, LINCS, STRING, OmniPath, the Therapeutic Target Database, DrugBank, and Pharos. The analysis scripts are available at https://github.com/jmjung83/NCES_framework. No additional restrictions apply beyond the access conditions of the respective databases.
