# Supplementary material for: NCES: A Cell-Specific Network-Augmented Essentiality Framework for Cancer Therapeutic Target Discovery
Source: Comput Struct Biotechnol J. 2026 Jul 17;35(1):0160. doi: 10.34133/csbj.0160 (PMC13376377; doi:10.34133/csbj.0160)
Supplement: Supplementary 1 — Figs. S1 to S5 Tables S1 to S11 [file csbj.0160.f1.zip › NCES_supplement_Figure(S1-S5).docx]

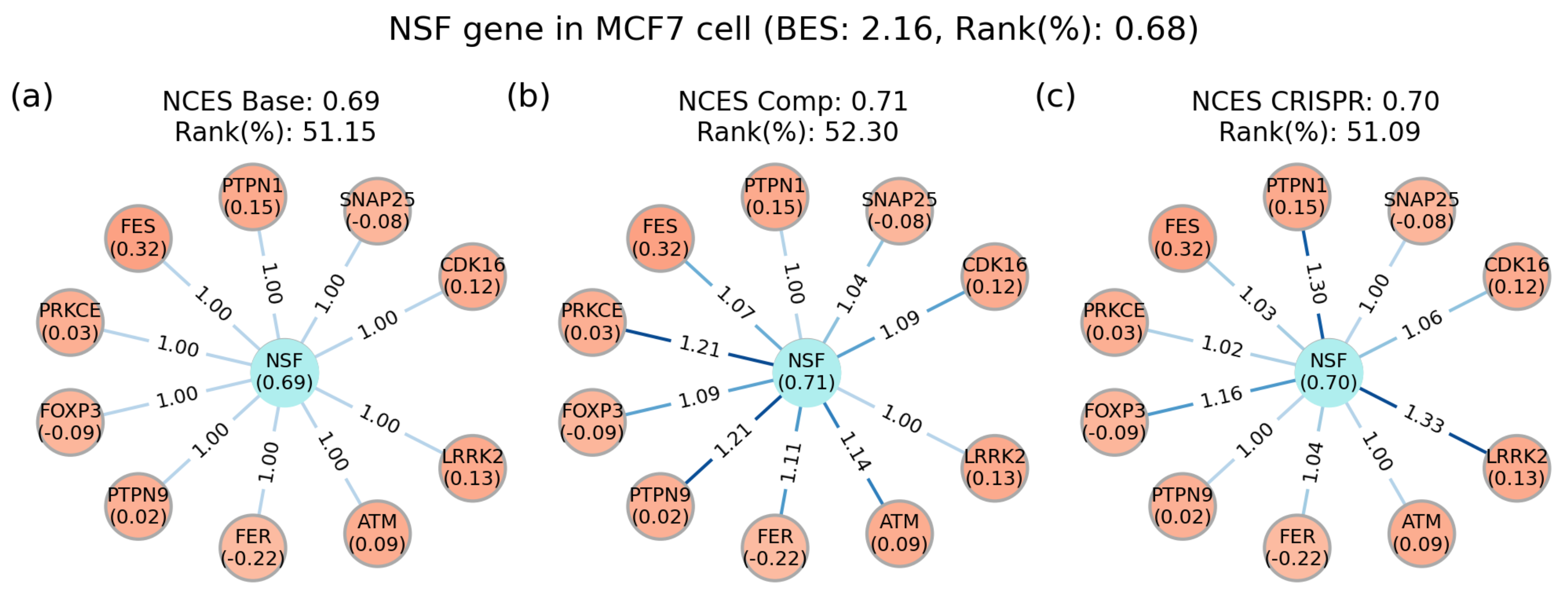


**Supplementary Figure S1. Network-based illustration of the three NCES variants for the NSF gene in MCF7**. Panels show the essentiality score (cyan central node) of NSF based on (a) NCES Base, (b) NCES Comp, and (c) NCES CRISPR. Neighboring genes are shown in orange, with their BES values indicated in the nodes, while edges are represented by blue lines, with numbers indicating the edge weights with the NSF gene. Unlike the DNAJB9 example in **Fig. 2**, none of the neighboring genes exhibits a strong essentiality signal, resulting in weak neighbor essentiality effects (NEEs) across all three NCES variants. Consequently, although NSF has a high BES of 2.16 (top 0.68%), its NCES values remain low at (a) 0.69 (top 51.15%), (b) 0.71 (top 52.30%), and (c) 0.70 (top 51.09%). This example illustrates that NCES can deprioritize highly essential genes when their functional neighbors provide limited network support. All NCES values shown were computed using the OmniPath-based PPI network and p = 0.8.


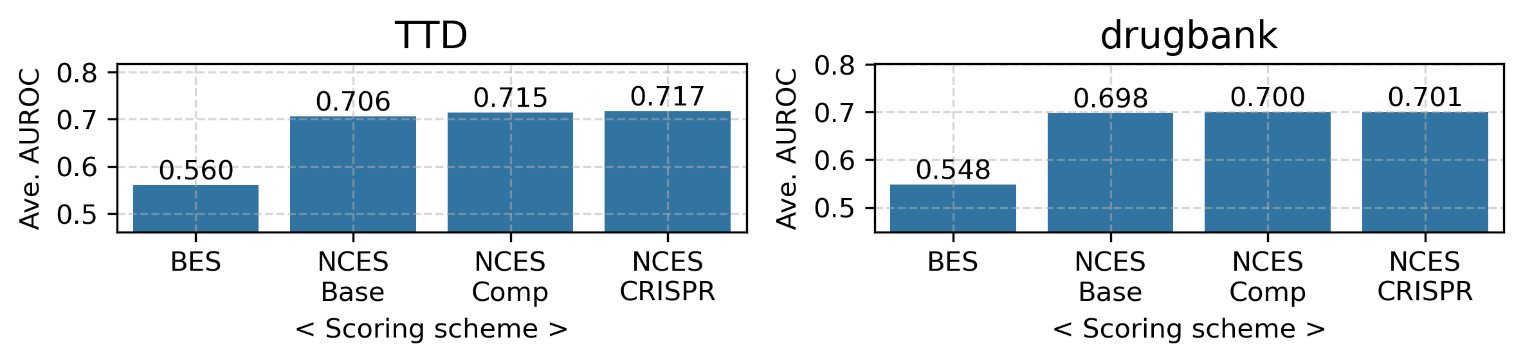


**Figure S2. Performance evaluation restricted to the druggable genome.** Average AUROC values across seven cancer cell lines for BES and NCES variants (Base, Comp, and CRISPR) evaluated using only druggable genes from the Pharos database (Tclin and Tchem; n = 2,608). Results for both TTD and DrugBank benchmarks show performance trends consistent with the primary analysis, with NCES-CRISPR achieving the highest predictive accuracy.


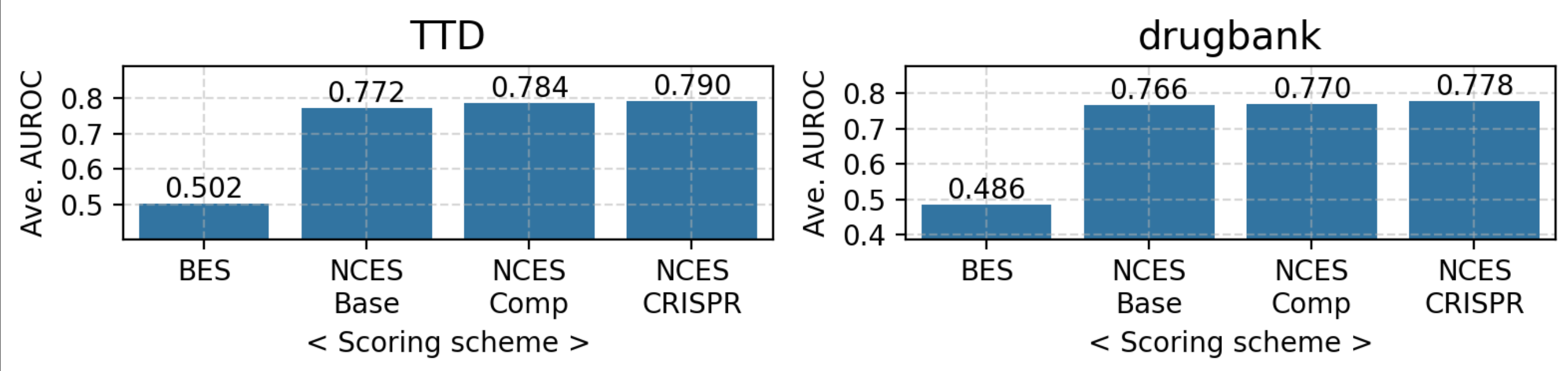


**Supplementary Figure S3. AUROC performance after excluding common essential genes.** Average AUROC values were recalculated after removing 1,827 common essential genes from the DepMap common essential gene set. NCES-CRISPR continued to achieve the highest performance for both TTD and DrugBank gold standard sets, and the overall ranking of methods remained unchanged.


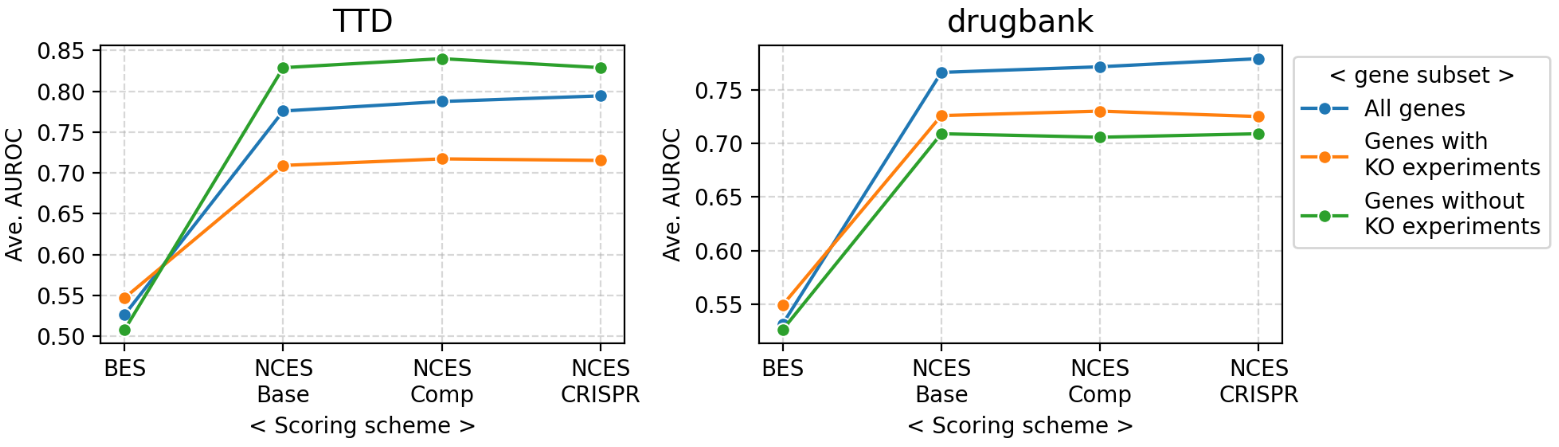


**Figure S4. Evaluation of NCES performance after controlling for LINCS CRISPR coverage bias.** Average AUROC values across seven cancer cell lines for BES, NCES Base, NCES Comp, and NCES CRISPR using TTD (left) and DrugBank (right) gold-standard sets. Performance was evaluated using three gene subsets: all genes (blue), corresponding to the original analysis; genes with available LINCS CRISPR knockout experiments (orange); and genes without available LINCS CRISPR knockout experiments (green). NCES consistently outperformed BES across all gene subsets. Notably, the performance advantage remained evident among genes lacking LINCS CRISPR knockout experiments, indicating that the predictive signal captured by NCES is not solely attributable to differential LINCS coverage and can be propagated through neighboring genes in the interaction network.


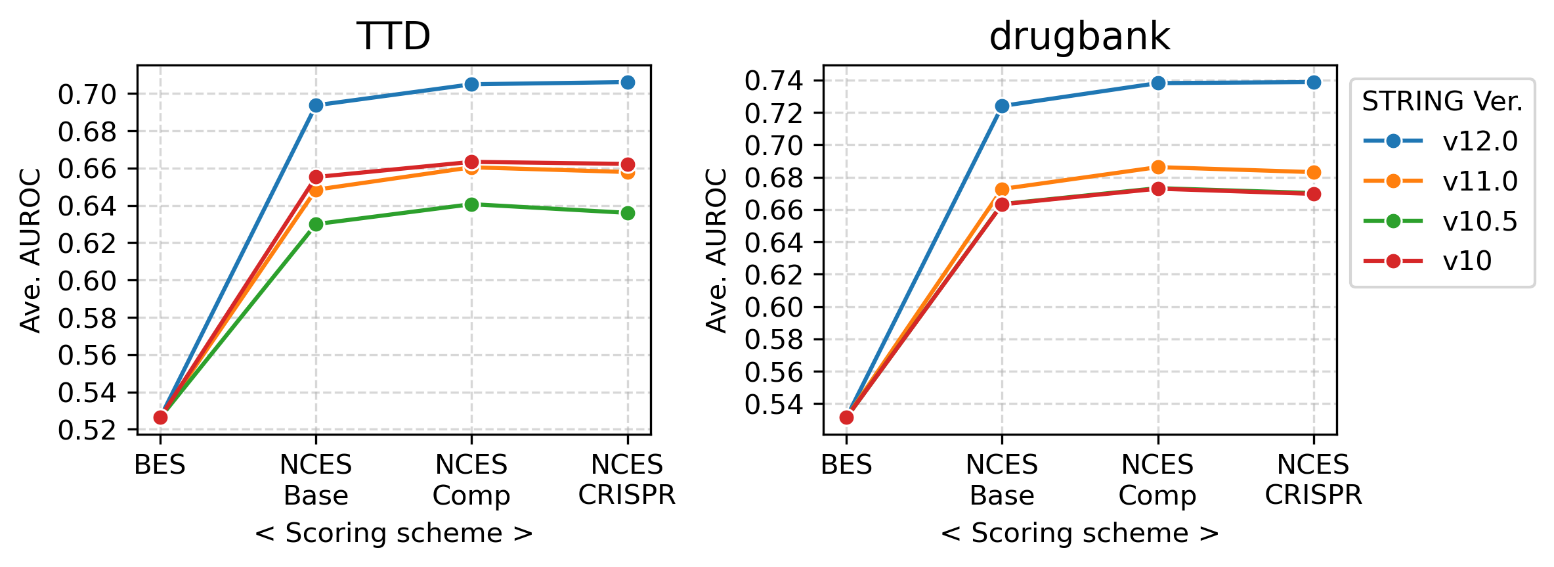


**Figure S5. Robustness of NCES performance across historical STRING versions.** Average AUROC values across seven cancer cell lines for BES and NCES variants evaluated using STRING v10 (2016), v10.5 (2017), v11.0 (2019), and v12.0 (2023). Results are shown for the TTD and DrugBank gold standards. NCES consistently outperformed BES across all STRING versions, and the relative performance ordering of the scoring schemes remained largely preserved.
